# Specific protein content of pools of plasma for fractionation from different sources: impact of frequency of donations

**DOI:** 10.1111/j.1423-0410.2010.01345.x

**Published:** 2010-10

**Authors:** R Laub, S Baurin, D Timmerman, T Branckaert, P Strengers

**Affiliations:** Central Department for Fractionation, Red CrossBrussels, Belgium

**Keywords:** albumin, donor remuneration, immunoglobulin, plasma donors, plasma fractionation, proteins

## Abstract

**Background and Objectives:**

Plasma pools for the production of human plasma medicinal products are distinguished according to the collection method (recovered or apheresis plasma) and the donor remuneration status. National regulations and the physical status of the donor determine the donation frequency and plasma volume per session. Relevant protein contents of different types of pools have not fully been compared.

**Materials and Methods:**

We compared the levels of total protein, 15 main relevant plasma protein markers, and *anti-B19* and anti-*Streptococcus pneumoniae* IgG in single-type pools of donations from different countries (Belgium, Finland, France, the Netherlands, Germany, United States). Both recovered plasma from non-remunerated donors and apheresis plasma from remunerated and non-remunerated donors were studied.

**Results:**

Pools from paid US high-frequency, high-volume plasmapheresis donors showed significantly lower total protein (−9%), albumin (−15%), total IgG (−24%), IgM (−28%), hemopexin (−11%) and retinol-binding protein (−10%) but higher C1-inhibitor, pre-albumin and C-reactive protein contents than pools from unpaid European Union (EU) or US whole-blood or plasmapheresis donors. In contrast to pools from compensated EU plasmapheresis donors, pools from unpaid whole-blood or plasmapheresis donors showed no significant differences, whatever the collection method or country. Reductions in specific protein contents correlated well with protein half-life.

**Conclusion:**

These results should be taken into account with regard to donor health management and protein recovery.

## Introduction

Human plasma-derived medicinal products are essential medicines for the treatment of patients with serious life-threatening chronic diseases and disorders [1]. Their production from plasma requires thoroughly appropriate donor selection, serological and microbiological donation screening, and pharmaceutical processing of plasma proteins. Plasma types can be classified according to the method of collection: recovered vs. apheresis (source) plasma, standard vs. hyperimmune plasma. Plasma types can also be distinguished according to the remuneration status of the donor (paid, compensated, or unpaid). Recovered plasma is produced by separating donated whole blood into cellular components and plasma. Source plasma is collected through apheresis, a process that takes only plasma from the donor while the cellular components are returned. Donation by plasmapheresis can be performed more frequently than whole-blood donation, as the body replaces the volume of donated plasma much faster than the volume of donated cellular components. Apheresis plasma can also be obtained as a by-product of platelets: in this case only the red cells are returned to the donor. This contribution to the manufacturing plasma pool is very low. Plasma derivatives are obtained through industrial-scale processing of a large number of pooled plasma donations. Plasma for fractionation must comply, in Europe, with monograph 01/2005:0853 of the European Pharmacopoeia. About 18 different therapeutic proteins are purified via a multi-step process including precipitations and/or chromatographic steps. With globalization, the demand for pharmaceutical plasma products, particularly intravenous immunoglobulin (IVIG) products, is growing at the rate of 3–5% per annum [2–4]. There is thus growing concern that a shortage of biological source material might occur, resulting in failure to meet the full demand for final products. This risk of shortage is stimulating discussions on the manufacturing yield of specific proteins and also on methods for increasing plasma supply, including high-frequency, high-volume plasma donations and the payment of plasma donors. Recognition of the importance of blood safety has led the World Health Organization (WHO) [5], the US Food and Drug Administration (FDA), the European Commission (EC), the Council of Europe [Council Europe 2008], the International Red Cross and Red Crescent Societies (IRCRCS), and the International Society of Blood Transfusion (ISBT) [6] to strongly discourage payment for whole blood. In Germany, monetary compensation of expenses is permitted for both apheresis and whole-blood donation [6]. The commercial for-profit fractionation industry, with its attendant plasma collection centres, relies primarily on paid donors, mostly living in the United States and Germany. The fractionation sector in China, emerging internationally, is also based on paid source plasma collection. In contrast, non-profit blood transfusion organizations in both Europe and the United States depend on the unpaid-donor network. Over the past decade in the United States, substantial changes have occurred with plasma source ‘rationalization’, i.e. a shift from independent plasma collection centres to concentration of their ownership in the hands of four major international fractionation companies [7].

The safety of paid- vs. unpaid-donor plasma is discussed at length in publications and at meetings, and remains controversial [8–11]. Plasma derivative safety relies on careful donor selection, extensive donation screening, efficient virus inactivation/removal steps included in the manufacturing process, and strict application of GMP rules at all stages of production. As a consequence of these measures, no transmission of infectious blood-borne diseases by plasma derivatives has been observed since 1997. The maximum volume of plasma to be donated and the donation frequency are regulated by national authorities and differ from country to country. For recovered plasma, the authorized volume ranges from 450 (±10%) to 500 (±10%) ml/donation, anticoagulant excluded. For apheresis plasma it ranges from 400 to 800 ml/donation, anticoagulant excluded. The donation frequency ranges from 3 to 5 times/year for whole blood and from 15 to 104 times/year for plasma.

Both source plasma and recovered plasma are known to be appropriate for use as starting material for the manufacture of plasma derivatives [1]. Differences have been observed as regards the yield. Apheresis plasma collected from donors undergoing frequent plasmapheresis contains lower levels of IgG but higher concentrations of clotting factors than plasma units produced by moderate serial plasmapheresis or from whole blood [1]. Whereas previous studies on intensive donor plasmapheresis have focused on donor safety, few have considered the composition of the collected plasma itself. In this study, we have compared the levels of various plasma proteins in plasmas for fractionation obtained by pooling of donations from either compensated (Group III) or paid (Group IV) donors with the levels observed in plasmas collected from unpaid donors (Groups I and II). The donations were collected in different countries and each pool studied was composed of donations from a single country. The proteins studied were chosen because they are key physiological proteins (hemopexin, immunoglobulin A, transferrin, IgM), inflammatory proteins [α-acid glycoprotein, C-reactive protein (CRP)], nutritional index proteins [retinol-binding protein (RBP) and prealbumin] or therapeutic proteins (albumin, immunoglobulin G, C1-inhibitor).

We have further investigated in what way this information might contribute to better understanding of donor safety in relation to donor vigilance.

## Materials and methods

### Donors and plasma donations

All donations were taken from suitable donors between February 2006 and January 2008. Individual donations were tested. Whole-blood units and apheresis plasma were collected into anticoagulant-preservative solution according to the European Pharmacopoeia, FDA, and AABB guidelines. The plasmas were processed by established by authorities recognized local blood transfusion centres (BTCs) in the European Union (EU, specifically in Belgium, Finland, France, Germany and the Netherlands) and in the continental United States (10 different centres at different locations). Processing was performed routinely, according to the established local procedures and specifications and in compliance with national regulations. Recovered and source plasma donations were tested and found to be non-reactive for anti-HIV1/2 antibodies, anti-HCV antibodies and HBsAg. Screening for anti-HTLVI/II antibodies was performed in Finland, the Netherlands, France and the United States according to national regulations. Nucleic acid technology (NAT) was used to test all donations for HIV1/2-RNA and HCV-RNA. Donations were also screened for HBV-DNA in Finland, the Netherlands, Germany and the United States. West Nile Virus was tested by NAT in US recovered plasma. Usually the donations destined for fractionation were additionally screened for Erythrovirus genotypes 1 & 2 (parvovirus) B19 DNA and HAV RNA on mini-pools. When the mini-pools were found positive, they were deconstructed and the positive donations rejected. The limit is < 10^4^ IU B19/ml in the manufacturing plasma pools (European Pharmacopoeia) [12].

The donors were healthy, voluntary and motivated. In Europe, they were mostly unpaid, in accordance with the Council of Europe criteria [13], or compensated [6]. In the United States, they were unpaid (recovered plasma collected at one BTC) or paid (source plasma from plasmapheresis centres). The total plasma unit volume per donation ranged from 280 to 320 ml for recovered plasma and from 581 to 814 ml for source plasma. All plasma donations were stored frozen at < −20 to −30°C within 24 h of collection and during shipment, and the temperature was monitored continuously. All donations were stored at −30°C at CAF-DCF. Table 1 shows the donors’ respective countries and remuneration statuses, the number of batches analysed, and the mean donation volume.

**Table 1:** Donors’ respective countries, number of batches and mean donation volume

Group	Remuneration	Method collection plasma	Number batches	Mean plasma volume per donation (ml)
**Group I**				
Finland	Unpaid	Recovered	6	288 ± 1^a^
France	Unpaid	Recovered	3	320 ± 7
Germany	Unpaid	Recovered	2	306 ± 1
The Netherlands	Unpaid	Recovered	10	318 ± 2
	Unpaid	Source	10	634 ± 5
Belgium	Unpaid	Recovered	10	280 ± 2
		Source	10	581 ± 7
**Group II**				
United States	Unpaid	Recovered	5	317 ± 14
**Group III**				
Germany	Compensated	Source	8	657 ± 95
**Group IV**				
United States	Paid	Source	41	814 ± 13

Taking the plasma density (*d* = 1·024) in account, the volume per donation for each batch is calculated as follows:

Total donations weight per batch – container weight/number of donations per batch.

a Mean of all batches ± standard deviation.

### Plasma pools and fractionation

All plasma pools were produced at the CAF-DCF (Brussels, Belgium) plant according to a modified Cohn-Oncley process adapted to its production. Briefly, each starting plasma pool (800–2700 l) for fractionation was produced from only recovered plasma or only source plasma from only one country. After the bags/bottles were cut, the plasma was extruded, thawed (1 ± 0·5°C) and centrifuged (Wesfalia BK28, Château Thierry, France) in a continuous process. This step yielded the first homogeneous plasma pool (the cryosupernatant) and a cryoprecipitate rich in Factor VIII.

All plasma pools were tested again for viral markers as described earlier and found to be non-reactive. All plasma pools complied with the specific monographs of the European Pharmacopoeia 5.0 [14].

A series of 50-ml cryosupernatant samples were taken from each batch and immediately stored at −80°C.

### Biochemical analyses

#### Total Protein

Total protein concentration was determined by the Biuret method. The standard was an 8% human albumin provided by Sanquin, Amsterdam, the Netherlands.

### Specific plasma protein analyses

The concentrations of albumin (HSA), α1-acid-glycoprotein (AGP), C1-inhibitor (C1-INH), hemopexin (HPX), immunoglobulin G (IgG) and IgG subclasses (IgG1, IgG2, IgG3, IgG4), immunoglobulin A (IgA), RBP, transferrin (TRF), prealbumin (PREALB), CRP and IgM were measured by nephelometry with a BN ProSpec apparatus (Dade Behring, Marburg, Germany). A calibrated in-house plasma pool standard was included in each run. The intra- and inter-run coefficients of variation (CVs) were < 6%, except for RBP and C1-INH, where the CV was about 10%.

### Specific anti-parvovirus B19 antibodies

After thawing, the homogeneous plasma pools (cryosupernatants) were first diluted 200-fold in the kit buffer. Total specific antibodies against the VP2 capsid protein of parvovirus B19 were measured by means of the ‘Parvovirus B19 IgG ELISA’ (Biotrin, Dublin, Ireland) according to the supplier’s instructions. The assay was calibrated with the WHO International standard 01/602 for anti-parvovirus B19 (NIBSC, South Mimms, UK).

### Specific anti-pneumococcal antibodies

#### Total anti-pneumococcal antibodies

Plasma pool samples were first diluted 100- to 300-fold in 8 mm Tris–HCl (pH 7·4) buffer containing 0·5% casein. Total specific anti-pneumococcus antibody determinations were performed with an enzyme-linked immunosorbent assay (ELISA) (ELIZEN; Zentech, Liège, Belgium) according to the supplier’s recommendations. The coating antigen was a mix of the 23 most frequent pneumococcus polysaccharides (serotypes 1, 2, 3, 4, 5, 6, 7F, 8, 9N, 9V, 10A, 11A, 12F, 14, 15B, 17F, 18C, 19F, 19A, 20, 22F, 23F, 33F). The cryosupernatant dilutions were first pre-incubated for 1 h at 37°C in the presence of 10 μg/ml C-polysaccharide (C-PS) (Statens Serum Institute, Copenhagen, Denmark) to increase the specificity of the test according to the WHO manual [15]. The FDA/CBER 89SF-5 pneumococcal reference serum was used as standard [16].

#### Serotype-specific anti-pneumococcal antibodies

To measure the concentrations of IgG directed against serotypes 10A, 18C, and 19A, the plasma was first mixed with C-PS absorbent and with the rare serotype 22F polysaccharide (LGS-ATCC Standards; Teddington, UK), according to the WHO manual [15]. The 22F antigen was added to avoid measuring cross-reacting antibodies capable of recognizing different pneumococcal serotypes. After a 60-min pre-incubation, 100 ml of each plasma dilution was added to microwells of a microtiter plate (MaxiSorp; Thermo Fisher, Roskilde, Denmark) coated with the appropriate individual serotype polysaccharide (0·1 μg/well). After 1 h at 37°C, the wells were washed with PBS-1% Tween 20 (pH 7·4). After washing, the serotype-specific antibody bound to the ELISA plate was detected with peroxidase-conjugated rabbit anti-human IgG antibody, followed by addition of the substrate tetra-methylbenzidine (Sigma Life Science, Bornem, Belgium). After a 30-min incubation, the reaction was stopped with 1 m H_2_SO_4_ and the optical density of each well was measured at 450 nm with a Microplate reader 3550 (Biorad, Gent, Belgium). By comparing the optical density in each sample well with the optical densities of wells containing the standard (human anti-pneumococcal reference serum, FDA/CBER batch 89SF-5), the level of antibody in the plasma pools was calculated.

### Data and statistical analyses

All data management/analysis was carried out with Excel and GraphPad Software, La Jolla, CA.

The specific anti-B19 and total anti-pneumococcus antibody concentrations were analysed following logarithmic transformation. Results are expressed as geometric means with standard deviations.

Statistical significance was evaluated with Student’s *t*-test. Differences with *P*-values below 0·05 were considered significant and ones with *P*-values below 0·0001 were considered highly significant.

## Results

Plasma types can be distinguished according to the method of collection (recovered or apheresis plasma), the donor remuneration status (paid, compensated or unpaid donors), and according to whether the plasma is polyvalent or hyperimmune regarding antibody content. In the present study, we compared the plasma protein compositions of recovered plasma and source plasma in fractionation pools, each pool being made with donations collected within a single country.

### Donation plasma volume and anticoagulant contribution

In the EU, the mean donation volume per unit was 302 ± 18 ml for recovered plasma and 608 ± 37 ml for source plasma (Group I donors) (Table 1). The mean donation volume collected from German Group III donors was 657 ± 95 ml source plasma/unit. In the United States, the mean source plasma donation volume per unit was 814 ± 13 ml, i.e. 34% higher than for Group I donors and 24% higher than for Group III donors.

Depending on the type of collection, different anticoagulants are used. In this study, all the recovered plasma donations contained the same anticoagulant, citrate-phosphate dextrose used at the same concentration (14/100 ml collected whole blood). For apheresis plasma donations, in most cases, the anticoagulant used was 4% sodium citrate in a 1:16 (6·3 ml anticoagulant for 100 ml blood) ratio. Some apheresis donations collected in the Netherlands and Belgium included the anticoagulant citrate-dextrose A (ACDA) (9–11 ml anticoagulant/100 ml blood). Taking in account the haematocrit for calculation and experimental data (H. Vrielink, J. Carlisle; personal communication), it results that apheresis plasma is less diluted than recovered plasma.

### Protein distribution between cryoprecipitate and cryosupernatant

The process of plasma thawing and centrifugation was continuous, with a flow rate of 500 l/h, leading to a first homogeneous plasma pool, the cryosupernatant and a cryoprecipitate. As expected, 98% of the total protein and 99% of the specific plasma proteins analysed were found in the cryosupernatant (data not shown).

### Protein contents of pools of EU recovered plasma vs. EU source plasma (Group I)

Recovered and source plasma donations were collected from Group I donors in five European countries (Finland, France, Germany, the Netherlands, and Belgium). Each plasma pool was produced with only one type of plasma donation from a single country. Thirty-one plasma pools were assembled with recovered plasma and 20 with source plasma (Table 1). The studied parameters were ‘total protein’, albumin (HSA), total IgG, IgM, IgA, TRF, HPX, α1-glycoprotein (AGP), RBP, C1-inhibitor (C1-INH), pre-albumin (PREALB) and CRP. These parameters were selected because of the following reasons: albumin and IgG (in intramuscular, subcutaneous and IVIG) are major medicinal plasma-derived products as is C1-INH, an active anti-inflammatory serine protease inhibitor, effective in the complement, contact and clotting systems. The levels of IgG and IgM are considered to be indicators of the humoral immune status. TRF is a key protein in the iron transport system. AGP and CRP are relevant to acute-phase inflammation. HPX is a scavenger of the toxic heme released or lost by heme proteins such as haemoglobin. IgA and PREALB are the proteins studied in long-term plasmapheresis [17]. RBP, a protein with a short plasma half-life (12 h), and PREALB has also been advocated as indicators of protein status in nutritional assessment [18].

**Fig. 1:**
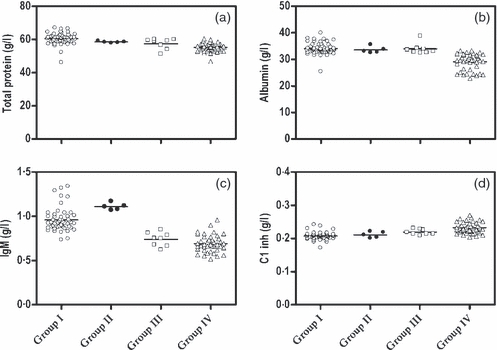
Total protein, albumin, IgM and C1-inhibitor contents of individual plasma pools collected from Group-I, Group-II, Group-III and Group-IV donors. Total protein (A), albumin (B), IgM (C) and C1-inhibitor (D) contents, expressed in g/l, were determined in individual batches according to Materials and Methods. Each plasma pool was produced from one type of plasma: Group I donors (o) (51 batches), Group II donors (•) (five batches), Group III donors (□) (eight batches) and Group IV donors (Δ) (41 batches). Each symbol represents one individual batch. The horizontal line (-) represents the mean of all batches of the specified type.

The individual data for each batch are presented in Fig. 1 for the markers ‘total protein’, HSA, IgM and C1-INH. Table 2 shows that the contents in total protein and 11 different proteins were very reproducible from batch to batch for recovered and source plasma. For most markers, batch variability was always < 10%, but it exceeded 17% for CRP.

**Table 2:** Compared protein contents of pools of EU recovered plasma, EU source plasma and US recovered plasma

	Content (g/l) in pools made from		
Markers	Group IRecovered plasma*n* = 31A	Group ISource plasma*n* = 20B	Group IIRecovered plasma*n* = 5C
Total protein	60·95 ± 2·35^a^	59·89 ± 4·66	58·43 ± 0·47
Albumin	33·92 ± 1·69	34·26 ± 2·97	33·56 ± 1·24
Total IgG	8·56 ± 0·61	8·32 ± 0·58	8·41 ± 0·32
IgM	0·95 ± 0·13	0·98 ± 0·14	1·11 ± 0·04
IgA	1·62 ± 0·21	1·65 ± 0·22	1·67 ± 0·18
Transferrin	2·22 ± 0·16	2·24 ± 0·22	2·30 ± 0·07
Haemopexin	0·70 ± 0·05	0·70 ± 0·07	0·73 ± 0·03
α_1_ glycoprotein	0·66 ± 0·03	0·68 ± 0·05	0·77 ± 0·01
Retinol-binding protein	0·03 ± 0·01	0·03 ± 0·01	0·03 ± 0·01
C_1_ inhibitor	0·21 ± 0·01	0·21 ± 0·02	0·21 ± 0·01
Prealbumin	0·19 ± 0·02	0·20 ± 0·04	0·19 ± 0·01
C-reactive protein	1·64 ± 0·28	1·85 ± 0·28	2·18 ± 0·22

Pools (*n* = 31) of EU Group I (as defined in Table 1) recovered plasma were compared with pools (*n* = 20) of EU Group I source plasma (columns A and B). Total protein and specific protein concentrations were determined as described under Materials and Methods. Additionally, 5 pools of recovered plasma (Group II) collected in the United States (unpaid donors) (C) were compared with recovered plasma collected in the EU (A). Differences proved to be non-significant for each marker (*P* > 0·05).

a The results are expressed as means of all batches ± standard deviation.

Table 2 also shows that no difference (*P* > 0·05) was observed for any tested protein marker between plasma pools made with recovered plasma and source plasma. Moreover, no difference (*P* > 0·05) was found between plasmas collected in the different EU countries (datanot shown). These data were therefore combined (Group I) for subsequent comparison with US plasma pools.

### Pools of EU plasma (Group I) vs. US recovered plasma (Group II)

Five individual pools of recovered plasma collected in the United States (Group II) were analysed for the same 11 protein markers (see Table 2). Again a great homogeneity of total and specific proteins was observed from batch to batch (except for RBP). No significant differences were detected between EU plasma (Group I) and US recovered plasma (Group II). These results seem to rule out any significant differences in plasma protein content between donations collected in countries with ethnically different populations.

### Pools of US Group IV source plasma vs. EU Group I plasma pools

**Fig. 2:**
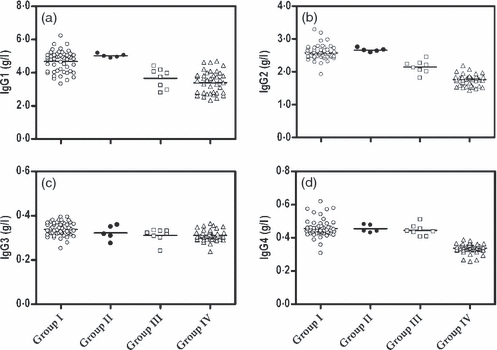
IgG subclass contents of individual plasma pools collected from Group-I, Group-II, Group-III and Group-IV donors. IgG1 (A), IgG2 (B), IgG3 (C); and IgG4 (D) contents, expressed in g/l, were determined in individual batches by nephelometry (see Materials and Methods). Each plasma pool was produced from one type of plasma: Group I donors (o) (51 batches), Group II donors (•) (five batches), Group III donors (□) (eight batches) and Group IV donors (Δ) (41 batches). Each symbol represents one individual batch. The horizontal line (-) represents the mean of all batches of the specified type.

The results are shown in Tables 3 and 4 and in Figs 1 and 2.

**Table 3:** Comparison of total protein and specific plasma protein contents in plasma pools collected from Group I and Group IV donors (mean ± SD)

	Content in g/l in donations			
Protein (g/l)	Group I*n* = 51A	Group IV*n* = 41B	%Variation^a^ C	*P*-value D
Total protein	60·46 ± 3·46^b^	55·20 ± 2·60	−9	< 0·0001
Albumin	34·05 ± 2·24	29·05 ± 3·08	−15	< 0·0001
Total IgG	8·48 ± 0·61	6·49 ± 0·51	−24	< 0·0001
IgM	0·96 ± 0·13	0·69 ± 0·09	−28	< 0·0001
IgA	1·64 ± 0·22	1·54 ± 0·18	−6	< 0·05
Transferrin	2·23 ± 0·18	2·06 ± 0·15	−7	< 0·0001
Haemopexin	0·70 ± 0·05	0·62 ± 0·06	−11	< 0·0001
α_1_ glycoprotein	0·67 ± 0·04	0·65 ± 0·07	−2	> 0·05
Retinol-binding protein	0·03 ± 0·01	0·03 ± 0·01	−10	< 0·05
C_1_ inhibitor	0·21 ± 0·01	0·232 ± 0·02	+12	< 0·0001
Prealbumin	0·19 ± 0·03	0·21 ± 0·02	+9	< 0·0001
C-reactive protein	1·72 ± 0·29	2·08 ± 0·67	+21	< 0·05

Batches of plasma pools collected from Group I donors (as defined in Table 1) (*n* = 51) (A) were compared with plasma pools from Group IV donors (*n* = 41) (B). Total protein was estimated by the Biuret method. Specific protein concentrations were measured by nephelometry (see Materials and Methods).

a The per cent variation of each marker, shown in column C, is expressed with respect to the Group I plasma pool value (column A) taken as 100% for each protein marker.

b The data are expressed as means ± standard deviations. *P* < 0·05 is considered significant. *P* < 0·0001 is considered highly significant (column D).

Forty-one plasma pools produced with US source plasma (Group IV) were individually analysed for their content in total protein and in 11 different specific proteins. The results were compared with those obtained for 51 EU plasma pools (Group I).

#### Content in main plasma proteins

The results are shown in Table 3 and Fig. 1. The total and specific protein contents did not vary much from batch to batch produced with Group IV donations. Except for AGP, both the total protein concentration and the specific plasma protein concentrations were significantly lower in the US source plasma pools, but to different extents. On the average, total protein was 9% lower than in EU plasma, RBP was 10% lower, and TRF was 7% lower. Although statistically significant, the difference found for IgA between the two types of plasma pools can be considered negligible (−6%).

Much lower in the US source plasma pools were the HPX (−11%), albumin (−15%), IgM (−28%) and total IgG (−24%) contents. Analysis of the individual data for the 41 US batches indicated that most of the batches contained total IgG levels ranging from 5·67 to 7·84 g/l, with seven batches (17%) containing < 6 g/l (Fig. 1). In contrast, in 98% of the EU batches, the total IgG concentration ranged from 7·11 to 9·82 g/l. The albumin content in the US batches ranged from 22·8 to 33·3 g/l, with 22 batches (53%) containing < 30 g/l. Except for one batch, the EU batches had an albumin concentration ranging from 31·4 to 40 g/l. The IgM content in US paid donation pools ranged from 0·52 to 0·96 g/l in contrast to the US Group II plasma (range 1·07–1·17 g/l) and EU Group I plasma (range 0·74–1·34 g/l).

The C1-INH, PREALB and CRP contents were significantly higher, however, in the US batches than in the EU batches (respectively, 12%, 9% and 21% higher).

#### γ-immunoglobulin subclasses

To determine whether IgG subclasses were affected by the lower total IgG amount in the US plasma pools (Group IV), the IgG1, IgG2, IgG3 and IgG4 concentrations were measured and compared in the unpaid US (Group II) and EU batches (Group I). As the total IgG recovery calculated by summing the results for the individual IgG subclasses exceeded 90% of the independently measured total IgG, further comparison was possible. No significant difference was found between donations collected from EU (Group I) and US Group-II donors. In the US plasma batches (Group IV), the IgG3 concentration was only 8% lower but the IgG1 and IgG4 concentrations were respectively, 27% and 26% lower (Table 4). A striking difference was found for IgG2, whose average concentration in the US plasma batches (Group IV) reached only 70% of that measured in the EU plasma pools. Figure 2 shows the individual data for all pools.

**Table 4:** Immunoglobulin G subclass contents of plasma pools collected from Group I, II, III and IV donors

	Donors			
IgG subclass (g/l)	Group I*n* = 51	Group II*n* = 5	Group III*n* = 8	Group IV*n* = 41
**IgG1**	4·67 ± 0·63^b^	5·01 ± 0·11	3·66 ± 0·60	3·40 ± 0·66
% Variation^a^		+7%	−22%	−27%
*P*-value		> 0·05	< 0·0001	< 0·0001
**IgG2**	2·57 ± 0·22	2·66 ± 0·06	2·14 ± 0·19	1·80 ± 0·17
% Variation		+3%	−17%	−30%
*P*-value		> 0·05	< 0·0001	< 0·0001
**IgG3**	0·34 ± 0·03	0·32 ± 0·03	0·31 ± 0·03	0·31 ± 0·02
% Variation		−5%	−8%	−8%
*P*-value		> 0·05	< 0·05	< 0·0001
**IgG4**	0·46 ± 0·05	0·45 ± 0·02	0·44 ± 0·03	0·34 ± 0·03
% Variation		−1%	−2%	−26%
*P*-value		> 0·05	> 0·05	< 0·0001

IgG subclass levels were determined in Group II (*n* = 5), Group III (*n* = 8), and Group IV (*n* = 41) plasma pools and compared with those of Group I plasma pools (*n* = 51).

a Percent variations with respect to Group I donation pool values taken as 100%.

b The results are means of the tested batches ± SD. IgG subclass contents were measured by nephelometry. *P* < 0·0001 is considered highly significant.

#### Content in specific antibodies against parvovirus B19 and Streptococcus pneumoniae

To evaluate the pathogen-neutralizing capacity of the immunoglobulins, we measured the titres of antibodies recognizing a virus, the parvovirus B19, and a bacterium, *Streptococcus pneumoniae*.

The US batches (Group IV) showed a significantly (26%) lower concentration of anti-B19 antibodies than the EU batches (Group I). When the titres were divided by the total IgG concentration, however, there no longer appeared any difference (Table 5).

**Table 5:** Anti-B19 and anti-*Streptococcus pneumoniae* antibody concentrations in plasma pools obtained from Group I, II and IV donors

	Donors					
	Group IA		Group IIB		Group IVC	
Antibody specificity	Conc.	%	Conc.	% vs. A^a^	Conc.	% vs. A^a^
***Parvovirus B19***	*n* = 51				*n* = 41	
IU/ml	32·2 ± 3·7	100			23·9 ± 4·6	−26*
IU/g Ig	3·8 ± 0·4	100			3·7 ± 0·7	−3
***S. pneumoniae***						
Total serotypes	*n* = 51		*n* = 5		*n* = 41	
μg/ml	35·4 ± 2·8^b^	100	34·3 ± 2·7	+1	22·5 ± 0·6	−36*
μg/g Ig^c^	4·2 ± 0·3	100	4·1 ± 0·4	+1	3·5 ± 0·1	−17*
**Serotype 10A**	*n* = 10		*n* = 5		*n* = 31	
μg/ml	1·4 ± 0·2	100	1·8 ± 0·3	+28	1·0 ± 0·3	−29*
μg/g Ig	0·17 ± 0·02	100	0·2 ± 0·04	+17	0·16 ± 0·04	−6
**Serotype 18C**	*n* = 10		*n* = 5		*n* = 31	
μg/ml	1·1 ± 0·1	100	1·3 ± 0·2	+18	0·53 ± 0·2	−52*
μg/g Ig	0·13 ± 0·02	100	0·15 ± 0·03	+15	0·08 ± 0·03	−38*
**Serotype 19A**	*n* = 10		*n* = 5		*n* = 31	
μg/ml	3·3 ± 0·5	100	7·0 ± 2·8	+112*	2·8 ± 1·0	−15
μg/g Ig	0·39 ± 0·06	100	0·84 ± 0·35	+115*	0·44 ± 0·16	+13

Specific anti-B19 and anti-pneumococcus antibody contents were measured by means of a specific ELISA, as described under Materials and Methods, in pools produced with Group I (A), Group II (B) or Group IV plasma (C). The number of batches (*n*) analysed is indicated.

a Represents the per cent of variation with respect to the level determined for Group I plasma pools, taken as 100%.

b Results are expressed as means ± SD for all plasma pools of each type.

c Specific antibody contents expressed per g total immunoglobulin (see Table 3).

* *P*-value highly significant < 0·001.

The total specific anti-pneumococcal antibody titre was 36% lower in the US batches (Group IV) than in the EU batches (Group I) (Table 5). When the titres were divided by the total IgG concentration, a 17% difference remained. To investigate whether the effect was similar for antibodies against each specific serotype, the capacity to neutralize each of three serotypes (serotypes 10A, 18C and 19A) was evaluated individually. The anti-19A IgG concentration turned out to be similar in both plasma pools, but the US batches showed respectively, 29% and 52% lower anti-10A and anti-18C levels. The difference was not significant for anti-10A and anti-19A antibodies when the titre was divided by the total IgG concentration, but the difference remained significant (37%) for anti-18C.

Further studies were performed on five batches of recovered plasma collected from US donors (Group II) to see whether the reduced amount of specific anti-pneumococcal antibodies measured in US plasma pools (Group IV) might result from a different epidemiological distribution of serotypes between the US and the EU. Table 5 does show a difference for anti-serotype 19A antibodies, whose level was more than twice as high in US (Group II) vs. EU (Group I) plasma pools. No significant difference was observed for serotypes 10A and 18C.

### Variation of protein contents with regard to protein half-life and donation frequency

**Fig. 3:**
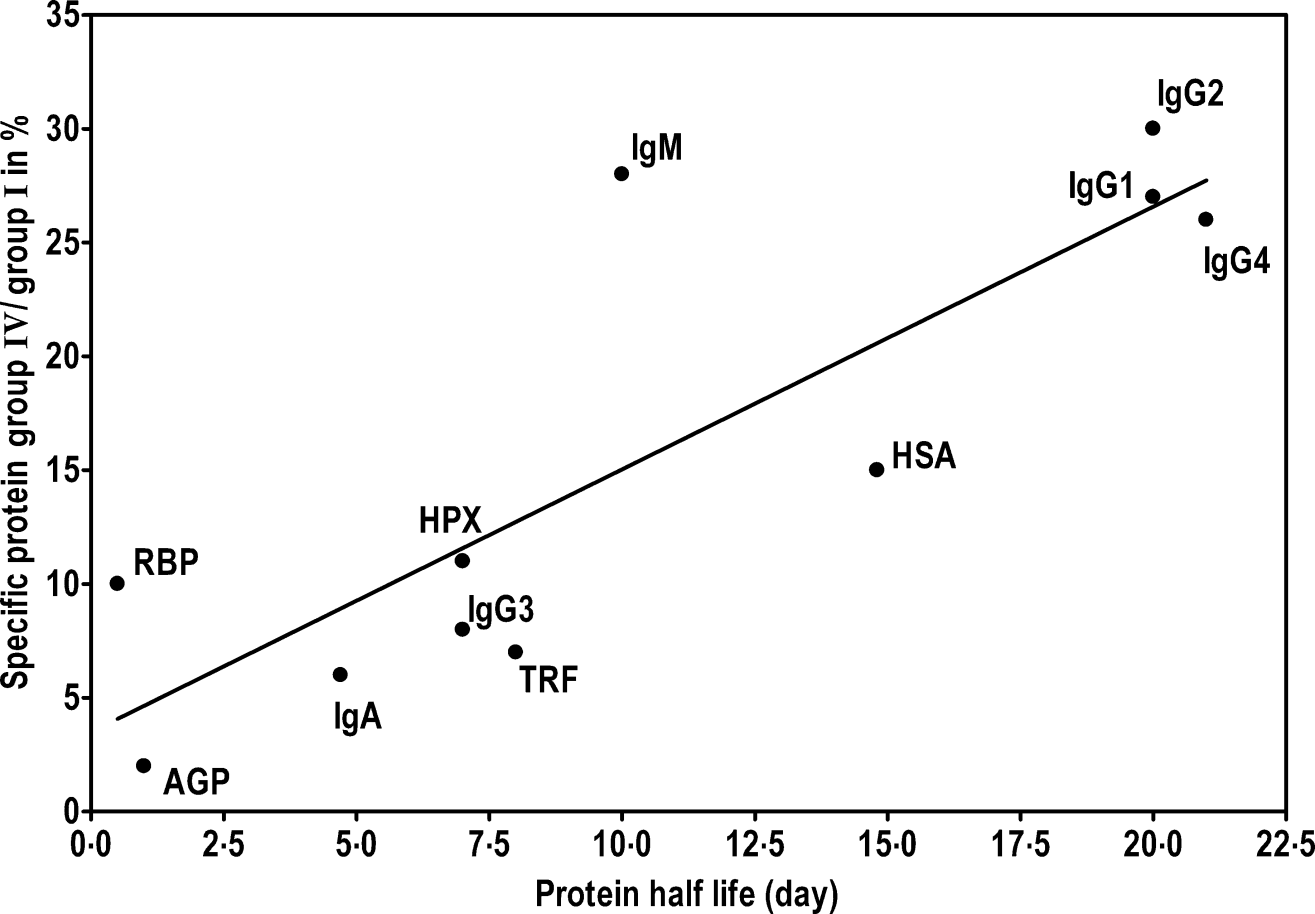
Specific plasma protein levels in Group II donors, expressed as percentages of the values obtained for Group I donors. The level of each protein measured in Group IV donor plasma pools is expressed as a percentage of that determined for Group I donor plasma pools and plotted as a function of the protein’s half-life (in days, as found in the literature [18–23]). The goodness of fit is 0·86 if the data for IgM are excluded and 0·71 if they are included.

The different levels of proteins in Group-IV vs. Group-I and Group-II donors suggest a relationship between the donation frequency and the donor capacity to replace these proteins. This replacement reflects the cumulated capacity of protein synthesis, depending strongly on the protein half-life, and the distribution between the vascular and extra-vascular compartments. Figure 3 shows that there exists, in general, a good linear correlation between the half-life of most of the proteins [18–23] and the decrease expressed as a percentage of the level measured in pools from Group I donors (*R*^2^ = 0·71 if IgM is taken into account and *R*^2^ = 0·86 if IgM is excluded).

### Pools of EU donor source plasma (Group III) vs. EU donor plasma pools (Group I)

To better understand the correlation between protein content and half-life, we studied eight batches obtained from Group III donors. In Germany, donors are permitted, as in the United States, to donate twice weekly but up to an annual maximum of 28·5 l (with anticoagulant) per year. The results shown in Table 4 and Figs 1 and 2 indicate that although the levels of four immunoglobulins (IgM, IgG1, IgG2 and IgG3) among the 11 markers were still significantly lower than in Group I donors, they were higher than in Group IV donors. The IgM decrease was 23% in Group III donors vs. 28% in Group IV donors. For IgG1 the decrease was 22% vs. 27%, and for IgG2 it was 17% vs. 30%. The total protein concentration was lower by a significant 6% in Group III donors (vs. 9% in Group IV donors). Only the C1-INH concentration was higher (6%) in Group III donors. The PREALB and CRP levels were in the range found for Group I donors.

The severely reduced plasma levels of IgM and IgG observed in Group III donors strongly suggest an impact of other, unknown factors, such as their distribution between the vascular and extravascular compartments.

## Discussion

As the demand for plasma products, mainly immunoglobulins, increases globally, and as new therapeutic indications emerge, it is necessary to find an adequate response to a strong and permanent demand for plasma. About 26·5 million litres of plasma for fractionation are collected worldwide, including 14·5 million in the US and 6·4 million in the EU [3]. Currently, 33% of all fractionated plasma is recovered plasma and 67% is obtained by apheresis.

We have compared recovered plasma from European (Group I) and US donors (Group II) and source plasma from European donors (Group I), German donors (Group III), and US donors (Group IV) to determine whether differences in the concentrations of the most relevant plasma proteins occur. The European countries included Belgium, Finland, France, Germany and the Netherlands. Each batch contained only one type of plasma from a single country. A first study, focusing on the European donor pools (Group I), compared the albumin, total IgG, IgA, IgM, transferrin, hemopexin, α1-glycoprotein, RBP, C1-inhibitor, prealbumin, CRP, and total protein contents of 31 pools of recovered plasma and 20 batches of source plasma. No significant difference was detected between recovered and source plasma, nor between countries where the plasma was collected. An impact of the anticoagulant volume according to the type of collection could be excluded, even though the anticoagulant-to-blood ratio of collected whole blood was higher than the ratio obtained in the apheresis process [24]. Our results show that plasmapheresis donation as performed currently in these European countries does not affect the specific plasma protein content compared to whole-blood donation. This finding encouraged us subsequently to consider all these batches together in our comparison with the US plasma batches. The Council of Europe (2008) [13] recommends a standard whole-blood donation of 450 ml + 10% excluding the anticoagulant (no more than 13% of the estimated total blood volume) and allows 4 (for females) to 6 (for males) standard whole-blood donations annually, with a minimum interval of two months. This period is amply sufficient to allow the plasma proteins to be synthesized and replaced and to reach again their physiological levels. A similar policy has been adopted by the FDA for whole-blood donations. A study of five different batches of recovered plasma from US donors (Group II) and the EU pools (Group I) revealed no differences between these batches as regards their total protein content or the levels of the 11 tested plasma proteins.

All 41 US plasma batches (Group IV) complied with the specific monograph for ‘Human plasma for fractionation’ (European Pharmacopoeia), with a total protein content above 50 g/l. A significantly (9%) lower total protein content was found, however, compared to the EU plasma batches (Group I). Lower levels of specific proteins were also found in the US batches (Table 2), but among these, three groups could be distinguished: 1) IgA and AGP, showing no significant difference; 2) TRF, RBP and HPX, with levels up to 11% lower; and 3) albumin, IgM and total IgG, with levels 15% to 28% lower.

The impact of long-term intensive plasmapheresis on plasma quality is well recognized and concerns a reduction of IgG, IgA, IgM, albumin and total protein [17,25,26]. However, the number of studies is limited because of the difficulty of conducting a prospective study on the safety of long-term plasmapheresis in donors [27]. Observed cases of decreased plasma protein levels following a plasmapheresis donation may result from differences in local regulations with respect to the amount of plasma allowed to be drawn, the time interval between two donations, and the monitoring system used. In 2004, in European Directive 2004/33/EC, the volume of a plasma donation increased from 650 ml (including the anticoagulant) to a maximum of 750 ml (excluding the anticoagulant) per procedure, with a maximum of 25 l (previously 15 l) per year, and with a 48-h interval between two donations. The previous more stringent rules are still in use in several EU countries (maximum 15 l/year). The present German Guidelines (2005) limit collection to 850 ml per session and up to 28·5 l/year, including the anticoagulant, with a minimum interval of 48 h between two donations. The FDA/CBER Guidelines for Automated Plasmapheresis (1992) set a limit based solely upon the body weight of the donor, from 690 ml (50–67·5 kg) to 880 ml (over 79 kg) including the anticoagulant, the frequency being limited to twice a week with a 48-h interval.

Hellstern *et al.* [28] compared 75 source plasma units (875 ml) collected in the United States with 75 units of German source plasma (720 ml). The median interval between two donations was 5 days in the United States and 14 days in Germany. Significantly lower total IgG, Factor V and Factor VIII levels were measured in the US donations. Bechtloff *et al.* [29] conducted a prospective trial on a small number of experienced plasmapheresis donors, who were asked to switch from a moderate to a more intensive plasmapheresis donation regime (750 ml/session and at least one per week) over a 3-year period. The donors showed significantly lower total protein, albumin and IgG levels than non-donors. The authors suggested that intensive plasmapheresis might cause a dysproteinemia pattern similar to that seen in the nephrotic syndrome. Recently, a prospective multicenter study on the safety of intensive plasmapheresis (720 or 850 ml per session) was performed, recruiting 2860 highly selected experienced donors used to donate 600 ml of plasma regularly over a 3-year period (SIPLA I) [26]. Despite a high dropout rate (about 75% of the total of donors), the results showed a significant reduction in the levels of IgG and total protein, and 14% of the donors had to be excluded because of a low plasma IgG level (< 5·8 g/l). This specific adverse event associated with automated donor plasmapheresis led to planning a second study (Siplan) including at least 60 000 donors with a minimum of 6·09 g/l IgG [30].

Our results reveal three groups of proteins whose levels appear to be differently affected in US source plasma pools. This finding suggests that these groups of proteins are reconstituted in the human body at different rates because of their different half-lives in plasma. Accordingly, a good linear correlation was seen between these groups of proteins and the half-lives of these proteins in plasma (Fig. 3). IgA and AGP have half-lives of < 5 days, and their concentrations were similar in the EU and US batches. Levels of the heme-binding protein HPX and of the iron-carrier protein TRF were respectively, 11% and 7% lower in the US batches, and their half-lives are respectively, 7 and 8 days. The two proteins HSA and total IgG have a low turnover rate (plasma half-lives: approximately 15 and 23 days, respectively). These data should be put in perspective with regard to the high frequency of donation and greater plasmapheresis volume allowed under FDA/CBER rules. The high frequency of collection and the high volume of maximum 800 ml twice a week does not allow a return to the normal level. Consequently, the concentration is 15–24% lower than expected.

To confirm this hypothesis, our study was extended to German donors (Group III), who are allowed to give a maximum of 28·5 l (including anticoagulant solution) per year. Eight pools containing source plasma from German donors were analysed for the same 12 markers. The results showed that only the IgM, IgG1, IgG2 and IgG3 contents were reduced. The strong reduction may be due mostly to less efficient antigenic stimulation resulting from the frequency of, and short intervals between donations.

Further analysis of the IgG subclasses revealed in US batches (Group IV) a severe deficit in IgG1, IgG2 and IgG4 (−27%, −30%, −26%, respectively), whose half-lives range from 20 to 21 days [20]. The slightly reduced IgG3 content might again be because of rapid turnover, based on a half-life of 7 days. This finding that the replacement of IgG3 is different from and faster than that of the other IgG subclasses should induce studies on its physiological function, which is unknown. The lower levels of specific anti-B19 and anti-pneumococcal antibodies reflect, partly in the latter case, the lower total IgG content of US source plasma batches (Group IV). Comparing the anti-B19 IgG titres of manufacturing plasma pools prepared from either source plasma or recovered plasma, Modrof *et al.* [31] found them to differ significantly, the titre being 28% higher in recovered plasma. Two of the main indications for total immunoglobulins (in IgG products such as IVIG) are primary and secondary immunodeficiency, as the corresponding patients are very susceptible to infectious diseases, notably caused by the various *S. pneumoniae* serotypes [32]. We have therefore measured titres of specific antibodies recognizing the serotypes 10A, 18C and 19A. Concentrations of specific anti-serotype-10A and anti-serotype-19A antibodies were lower in the US batches (Group IV) because of the lower total IgG content. As for the lower anti-serotype-18C titre, it cannot be attributed to an epidemiological difference between the two continents, i.e. to different pneumococcal serotype frequencies [33–35], as the titre of anti-serotype 18C was not significantly different in EU and US recovered plasma pools, in contrast to the titre of anti-serotype 19A. Serotype 19A is frequent in the US. Its higher frequency in the United States than in Europe may explain why the corresponding IgG was about twice as abundant in the US plasma batches.

Interesting is the 10% lower concentration of RBP in the US source batches. Because of its very short half-life (about 12 h), this specific retinol carrier protein, which belongs to the lipocalin family, is affected by inflammation. In recent years, plasma protein markers have been shown to be reliable indicators of nutritional status, in particular during specialized nutrition support intervention. RBP (plasma half-life: 12 h), TRF (half-life: 8 days) and albumin (half-life: 15 days) have been selected as nutritional deficiency indicators included in the Nutritional Risk Index, being representative, respectively, of proteins with very short, short, and long to extremely long half-lives [18]. Our results invalidate the view that the body replaces the donated plasma usually within 24–48 h if the donor keeps a healthy diet [36]. The low plasma protein level observed in US plasma donors (Group IV) might not be considered indicative of any disease, but it shows the presence of a not completely healthy condition compared to non-donors or Group I donors.

In the present study some proteins, on the contrary, were found at higher concentrations in the US batches (Group IV) than in the EU batches (Group I): C1-INH and PREALB were respectively, 12% and 8·9% higher. CRP, a protein associated with inflammation, was 21% higher in US plasma but still below the pathological level.

Regarding donor vigilance, the relatively low level of albumin in Group IV indicates that donors with an undiagnosed cardiovascular disorder, or at risk of developing one, should be rejected as high-frequency, high-volume donors to avoid oedema. Although our donor vigilance study did not focus on this aspect, Seidel (2009) [37], discussing red cell management during frequent plasmapheresis, underlined the little attention paid to the impact of blood loss for blood sampling and additional loss of residual blood in the bowl and tubing of the disposable set after the donor has been disconnected from the machine. Donors frequently have to be turned away from plasma donation because of a too-low haematocrit/haemoglobin level. Without flushing with saline at the end of the procedure, an additional loss occurs per annum of 1440 ml blood (equivalent to three whole-blood donations) in Germany (where plasma donors donate up to 40 times/year) and 3744 ml blood (equivalent to more than seven whole-blood donations) in the United States (where the donors may donate up to 104 times/year). This finding should be studied further to prevent high-frequency, high-volume plasmapheresis donors from donating unintentionally an excessive amount of cellular components on an annual basis.

## Conclusion

Our findings show that significant differences can be found in plasma protein levels in plasmas collected with different techniques and frequencies. Whether these significant differences have relevant health implications for the donors is questionable. Physiologically, the differences in albumin level between the EU plasma pool (Group I) and the US source plasma pool (Group IV) should be studied more carefully. In particular, the lower albumin level in the US source plasma donors (Group IV) might result in a lower osmotic pressure leading possibly to oedema in older donors, donors with an undiagnosed cardiovascular disorder, or donors at risk of developing one.

The need to obtain IgG for manufacturing IVIG is the driving force for high-frequency, high-volume source plasma collection. Yet the physiological replacement of IgG is relatively slow, and restoration of normal physiological levels takes time. This raises the question of whether it might be advisable to reduce the frequency so as not to jeopardize the humoral health status of the donor.

For rational plasma collection with a view to meeting the increasing demand for plasma products, it is necessary to conduct further studies on the physiological effects of drawing plasma from plasma donors, so as to avoid impairing the health status of donors.
